# Evaluation of the antimicrobial use in pigs in Japan using dosage-based indicators

**DOI:** 10.1371/journal.pone.0241644

**Published:** 2020-10-30

**Authors:** Reiko Abe, Hiroko Takagi, Kyoko Fujimoto, Katsuaki Sugiura

**Affiliations:** Department of Global Agricultural Sciences, Graduate School of Agricultural and Life Sciences, the University of Tokyo, Tokyo, Japan; Nitte University, INDIA

## Abstract

The use of antimicrobial agents in food-producing animals may lead to the emergence and spread of antimicrobial resistance in bacteria of animal origin. The use of antimicrobial agents in pigs in 2018 in Japan was evaluated in terms of the weight of active ingredient and number of defined daily doses (DDD), using annual sales data of veterinary antimicrobials sold for use in pigs. In addition, the use of antimicrobial agents in the Japanese pig sector in 2008 to 2017 was evaluated to determine whether or not there were any differences in temporal change pattern by use of different metrics. In 2018, 447 metric tons of active ingredient, corresponding to 77,379 × 10^6^ kg-days (Japanese DDD) and 34,903 × 10^6^ kg-days (European DDD) were sold. The proportion of the sales amount of sulfonamides, trimethoprim and lincosamides to the total sales amount was significantly different depending on the metric used. For most antimicrobial classes, the number of Japanese DDDs was greater than the number of European DDDs. These results indicate that the DDD-based metric, which is more reflective of the selective pressure of antimicrobials, is recommended for use in monitoring the antimicrobial use in pigs in Japan. The differences in the number of Japanese DDDs and European DDDs appear to confirm the need for Japanese DDDs.

## Introduction

Increased antimicrobial resistance in bacteria that cause infections in humans is a threat to public health. The use of antimicrobials in food-producing animals in the form of veterinary medicine and feed additives might lead to the emergence and spread of antimicrobial resistance in bacteria of animal origin. Currently 700,000 people die of resistant infections every year. If no proactive solutions are taken to reduce the rise of drug resistance, by 2050, some 10 million lives per year could be at risk from drug resistant infections [1]. Bacterial resistance arises through complex mechanisms, normally by mutation and selection, or by the acquisition of genetic information that encodes resistance from other bacteria [2]. Therefore, diminishing the selection pressure by reducing antimicrobial use is considered to be one of the important strategies to prevent and control the emergence and spread of antimicrobial resistance [2].

As in European countries, over half of the veterinary antimicrobials purchased in Japan are used in pigs [3–9]. Therefore, reducing the use of antimicrobials and the promotion of prudent use in pig production are important strategies to reduce selection pressure and thus to lower resistance rates.

There is no global consensus on the collection of antimicrobial use, data and reporting methods but many activities in this field are in progress [10]. Under the European Surveillance for Veterinary Antimicrobial Consumption (ESVAC) project of the European Medicines Agency (EMA), European countries routinely report total quantities of antimicrobials sold for use in food-producing animals as mg of active ingredient, adjusted by animal biomass (population correction unit: PCU) [11]. The authors have previously investigated the use of antimicrobial agents in food-producing animals in Japan in terms of mg of active ingredient sold per kg of biomass [8, 9]. This metric is simple to calculate and easy to understand. However, use of this metric might encourage favouring high potency antimicrobials given their lower mg quantity per dose [12].

In Denmark, the Netherlands and some other European countries and Canada, dosage-based indicators are used to monitor antimicrobial usage at the farm level [13–15]. Dose-based indicators have the advantage of making it possible to correct dosage differences between active ingredients and formulations and to measure developments over time, despite changes in which active ingredients are used [16]. In 2016, the EMA published the average defined daily dose (DDDvet) values for antimicrobial agents used in food-producing animals as a tool to facilitate the standardised collection and presentation of antimicrobial use among EU member states [17]. These values were defined by calculating the mean dose of antimicrobial products registered in nine EU member states.

To establish a monitoring system using an indicator based on daily dosage, the authors have previously assigned DDD values for 354 veterinary antimicrobial products approved and marketed for use in pigs in Japan [18].

The aim of this study was to assign Japanese DDD (DDDjp) values for each antimicrobial agent (active ingredient) based on the DDD values assigned to the products. Using these DDDjp values and DDDvet values, we evaluated the sales of antimicrobials agents destined for use in pigs in Japan in 2018 in terms of the number of Japanese and European DDDs. The use of antimicrobial agents in pigs in Japan from 2008 to 2017 was also evaluated to determine whether or not there have been differences in temporal change patterns when using these metrics.

## Materials and methods

### Antimicrobial sales data collection and calculation of the weight of active ingredient

Manufacturers and importers of veterinary antimicrobials in Japan are required, under the Regulations for Control of Veterinary Pharmaceutical Products (Ministerial Order No.3, 1961), to submit details of the sales quantity of veterinary antimicrobials to the Minister of Agriculture, Forestry and Fisheries each year. The data submitted must include the names of antimicrobial products, routes of administration, concentrations of the active ingredient in each product and the target animal species for which the products are used [19]. Annual antimicrobial sales data submitted in this way are compiled into a database by the National Veterinary Assay Laboratory of the Ministry of Agriculture, Forestry and Fisheries, which is available from their website [19]. We used the sales data from 2008 to 2018 and calculated the sales quantity of active ingredient sold for use in pigs by antimicrobial class and administration route.

### Assignment of Japanese Defined Daily Dose values for antimicrobial agents (DDDjp)

The DDDjp values were calculated using the DDD values that we previously assigned for 354 veterinary antimicrobial products approved and marketed for use in pigs in Japan [18]. The DDDjp values were calculated by averaging the DDD values of products if there were two or more products containing the same antimicrobial agent. For those antimicrobial agents that are used as active ingredient in products both for injection and oral administration, DDD values were assigned separately for each administration route. Likewise, for those that are used both in single substance and combination products, DDD values were assigned by averaging dosages of both the single substance and combination products. In other words, the average (arithmetic mean) of all DDD values of products for each combination of antimicrobial agent and administration route was used to assign DDDjp–e.g. benzylpenicillin/parenteral.

### Calculation of the number of defined daily doses

To calculate the number of DDDjps and DDDvets of each antimicrobial active ingredient, the amount of antimicrobial active ingredient sold each year from 2008 to 2018 was divided by the DDDjp and DDDvet of the corresponding antimicrobial active ingredient. The DDDvet values were available from the EMA website [17].

$$Number\;of\;DDDjps\;(kg‐days)=\frac{Weight\;of\;active\;ingredient(mg)}{DDDjp\;value\;(\frac{mg}{kg-day})}$$ Formula 1

$$Number\;of\;DDDvets\;(kg‐days)=\frac{Weight\;of\;active\;ingredient(mg)}{DDDvet\;value(\frac{mg}{kg・day})}$$ Formula 2

In calculating the number of DDDvets using formula 2, the DDDjp value was used for those antimicrobial ingredients for which DDDvet was not available.

The weight of active ingredient and the corresponding number of DDDjps and DDDvets were calculated in total, for the different administration routes (parenteral and oral) and for all antimicrobial classes.

### Classification of antimicrobial agents

The antimicrobial agents were classified into 13 groups based on the Anatomical Therapeutic Chemical classification system for veterinary medicinal products (ATCvet) proposed by the World Health Organization (WHO) [20]: tetracyclines; amphenicols; penicillins; sulfonamides; macrolides; lincosamides; aminoglycosides; pleuromutilins; cephalosporins; trimethoprim; polymyxins; quinolones; and others. The specific classification of antimicrobial agents and their DDDjp values used are presented in Table 1.

**Table 1:** Defined Daily Dose (DDD) values used for the evaluation of antimicrobials sold for use in pigs in Japan.

Antimicrobial class	Antimicrobial agent	Single substance or combination^a^	Administration route	DDDjp value	DDDvet value
				(mg/kg day)	(mg/kg day)
Tetracyclines	Oxytetracycline	Single	Parenteral	6.5	7.5
	Oxytetracycline_LA	Single	Parenteral	5.0	-
Amphenicols	Thiamphenicol	Single	Parenteral	20.0	75.0
	Florfenicol	Single	Parenteral	5.0	9.5
Penicillins	Ampicillin	Single	Parenteral	6.5	12.0
	Amoxicillin	Single	Parenteral	7.5	8.9
	Mecillinam	Single	Parenteral	3.8	-
	Benzylpenicillin	Single and combination	Parenteral	4.6	9.2^b^
	Aspoxicillin	Single	Parenteral	3.8	-
Cephalosporins	Cefazolin	Single	Parenteral	5.0	-
	Ceftiofur	Single	Parenteral	2.5	3.0
	Cefquinome	Single	Parenteral	1.5	1.9
Sulfonamides	Sulfadimethoxine	Single	Parenteral	60.0	30.0
	Sulfamonomethoxin	Single	Parenteral	70.0	-
	Sulfadoxine	Combination	Parenteral	30.0	14.0
Trimethoprim	Trimethoprim	Combination	Parenteral	6.0	3.0
Macrolides	Erythromycin	Single	Parenteral	4.5	21.0
	Tylosin	Single	Parenteral	6.0	13.0
	Tulathromycin	Single	Parenteral	2.5	-
	Mirosamycin	Single	Parenteral	5.0	-
	Tilmicosin	Single	Parenteral	10.0	-
Lincosamides	Lincomycin	Single	Parenteral	7.5	10.0
Aminoglycosides	Dihydrostreptomycin	Single and combination	Parenteral	24.6	16.1 ^b^
	Kanamycin	Single	Parenteral	15.0	28.0
	Kanamycin	Single	Topical	110.0	-
Quinolones	Enrofloxacin	Single	Parenteral	2.6	3.4
	Danofloxacin	Single	Parenteral	1.3	1.2
	Marbofloxacin	Single	Parenteral	2.0	-
	Orbifloxacin	Single	Parenteral	3.8	-
Pleuromutilins	Tiamulin	Single	Parenteral	10.0	12.0
Others	Fosfomycin	Single	Parenteral	15.0	-
Tetracyclines	Doxycycline	Single	Oral	9.0	11.0
	Chlortetracycline	Single and combination	Oral	9.6	24.8 ^b^
	Oxytetracycline	Single and combination	Oral	8.7	22.5 ^b^
Amphenicols	Thiamphenicol	Single	Oral	5.0	35.0
	Florfenicol	Single	Oral	1.5	10.0
Penicillins	Ampicillin	Single	Oral	8.0	30.0
	Amoxicillin	Single	Oral	6.5	17.0
	Benzylpenicillin	Combination	Oral	0.8	–
Sulfonamides	Sulfadimethoxine	Single and combination	Oral	43.2	28.5 ^b^
	Sulfamonomethoxin	Single and combination	Oral	31.1	22.2 ^b^
	Sulfamethoxazole	Combination	Oral	4.7	20.0
	Sulfadimidine	Combination	Oral	6.0	23.0
Trimethoprim	Trimethoprim	Combination	Oral	1.6	4.7
	Ormethoprim	Combination	Oral	2.7	–
Macrolides	Tylosin	Single	Oral	11.3	12.0
	Tilmicosin	Single	Oral	5.0	15.0
	Tylvalosin	Single	Oral	1.4	3.6
	Mirosamycin	Single	Oral	2.5	–
Lincosamides	Lycomycin	Single	Oral	5.1	7.6
Aminoglycosides	Streptomycin	Single and combination	Oral	10.5	13.7 ^b^
	Gentamycin	Single	Oral	0.6	1.4
	Kanamycin	Combination	Oral	4.2	–
	Apramycin	Single	Oral	4.0	9.0
	Fragiomycin	Combination	Oral	4.9	–
Quinolones	Norfloxacin	Single	Oral	7.5	–
	Orbifloxacin	Single	Oral	3.8	–
	Enrofloxacin	Single	Oral	1.9	–
	Ofloxacin	Single	Oral	7.5	–
	Oxolinic acid	Single	Oral	20.0	26.0
Pleuromutilins	Tiamulin	Single	Oral	6.2	9.7
	Valnemulin	Single	Oral	2.6	5.3
Polymyxins	Colistin	Single	Oral	4.8	5.0
Others	Bicozamaycin	Single	Oral	7.5	–

DDDjp: Japanese defined daily dose values.

DDDvet: European defined daily dose values assigned by the European Medicines Agency.

a: ‘Single’ indicate that the substance is used as an active ingredient in single substance products, and ‘combination’ indicates that the substance is used as an active ingredient in products containing two antimicrobial agents.

b: Antimicrobial agents for which different DDDvet values are assigned for single and combination products, DDDvet values are integrated into one value in this study by averaging the values for single and combination products.

–: The DDDvet value has not been assigned by the European Medicines Agency.

### Statistical analysis

The correlation between the number of DDDjps and the number of DDDvets for different antimicrobial classes was investigated using Spearman’s Rho test. Statistical analysis was conducted using Excel 2010 (Microsoft Corporation) and BellCurve for Excel ver. 3.00 (Social Survey Research Information Co., Ltd.) added to Excel.

## Results

### Antimicrobial sales amount for use in pigs in 2018

The antimicrobial agents sold for use in pigs in Japan was calculated to be 447 tons of active ingredients and 77,379 million DDDjps using Japanese DDD values, indicating that theoretically a total of 77,378 million kg-days of biomass were treated with antimicrobials in 2018. The number of DDDvets was 34,903 million, indicating that the number of DDDs was more than twice as large when calculated using DDDjp than when calculated using DDDvet (Table 2). When investigating the different administration routes by the number of DDDs, the number of DDDs using the oral route represented the largest proportion regardless of the metrics used.

**Table 2:** Antimicrobial sales amount in pig sector in Japan in 2018 grouped by different antimicrobial classes.

Antimicrobial class	Total			Parenteral			Oral		
	Weight of active ingredient(kg)	Number of DDDjps (1,000s)	Number of DDDvets (1,000s)	Weight of active ingredient(kg)	Number of DDDjps (1,000s)	Number of DDDvets (1,000s)	Weight of active ingredient(kg)	Number of DDDjps (1,000s)	Number of DDDvets (1,000s)
Tetracyclines	198,500	21,927,621	12,179,040	504	77,555	67,215	197,996	21,850,066	12,111,825
Amphenicols	16,938	8,998,572	1,389,823	882	131,627	65,407	16,057	8,866,945	1,324,417
Penicillins	45,076	6,375,912	2,281,352	4,264	647,610	367,102	40,812	5,728,302	1,914,251
Cephalosporins	601	240,465	200,387	601	240,465	200,388	0	0	0
Sulfonamides	60,111	10,848,221	2,801,214	910	18,541	32,573	59,201	10,829,680	2,768,641
Trimethoprim	10,038	6,158,234	2,175,033	52	8,679	17,358	9,986	6,149,555	2,157,675
Macrolides	29,279	8,239,295	2,998,211	461	135,676	116,433	28,817	8,103,619	2,881,778
Lincosamides	16,140	3,177,942	2,114,866	281	37,493	28,120	15,859	3,140,450	2,086,746
Aminoglycosides	19,458	2,439,260	2,120,200	911	36,970	56,687	18,547	2,402,291	2,063,513
Quinolones	1,880	458,731	436,325	720	261,554	239,165	1,160	197,176	197,161
Pleuromutilins	36,667	6,048,092	3,838,897	74	7,400	6,167	36,593	6,040,692	3,832,731
Polymyxins	11,829	2,464,430	2,365,853	0	0	0	11,829	2,464,430	2,365,853
Others	16	2,154	2,154	0	0	0	16	2,154	2,154
Total	446,534	77,378,935	34,903,359	9,660	1,603,572	1,196,614	436,874	75,775,362	33,706,745

DDDjps: Japanese defined daily doses; DDDvets: European defined daily doses.

**Fig 1:**
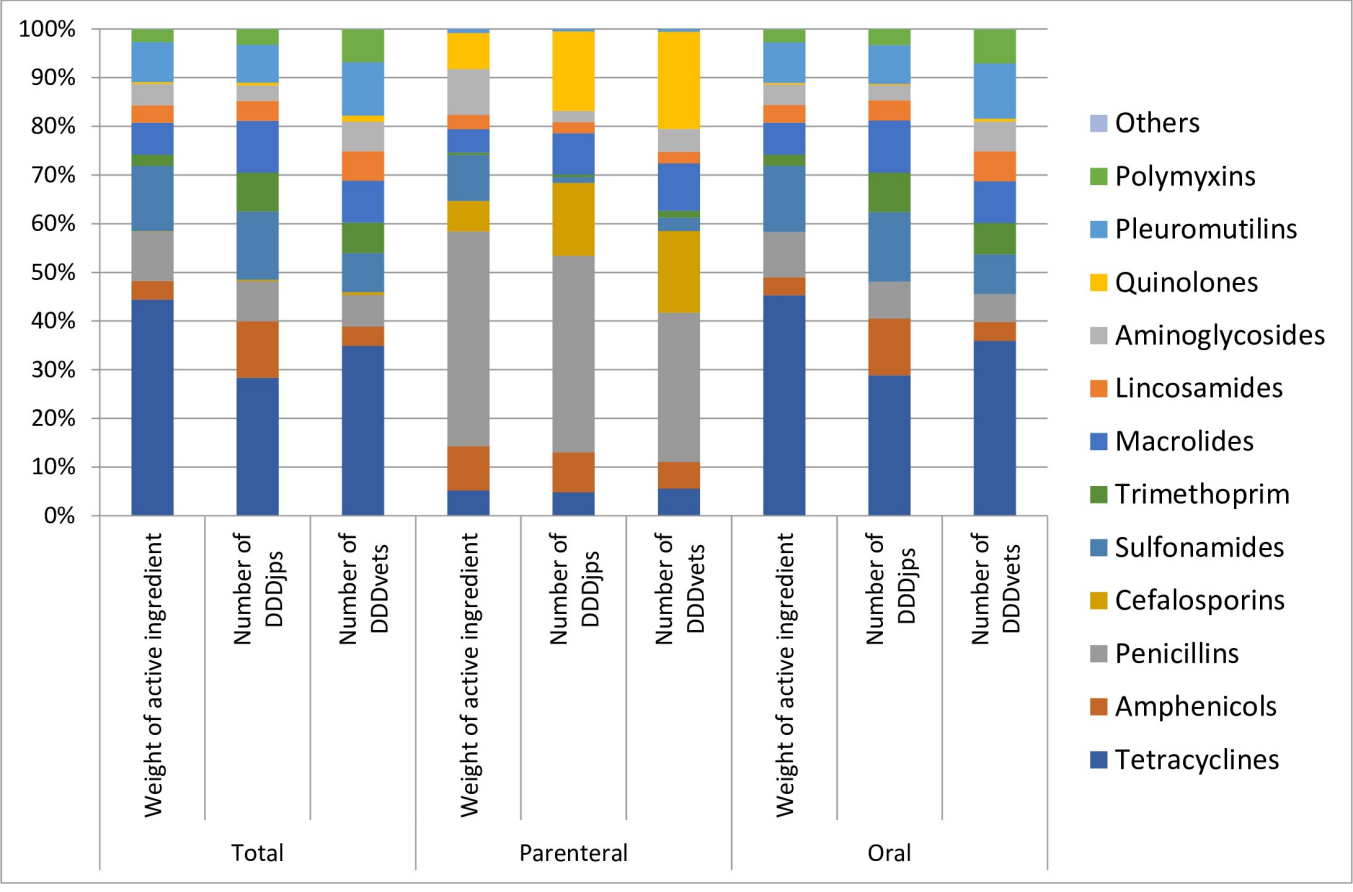
Relative distribution of antimicrobial sales in the pig sector in Japan in 2018 showing different antimicrobial classes according to administration route and metric.

Fig 1 provides the relative distribution of antimicrobial use between different antimicrobial classes by administration route measured either as the amount of active ingredient or as the number of defined daily doses (DDDjp and DDDvet).

### Antimicrobial sales for parenteral use in pigs in 2018

In terms of the weight of active ingredient, penicillins represented the largest proportion (4,264 kg, 44.1%) of the total usage, followed by aminoglycosides (911kg, 9.4%) and sulfonamides (910kg, 9.4%). In terms of the number of DDDjps, penicillins represented the largest proportion (648 million kg-days, 40.4%) of the total usage, followed by quionolones (262 million kg-days, 16.3%) and cephalosporines (240 million kg-days, 15.0%). In terms of the number of DDDvets, penicillins represented the largest proportion (367 million kg-days, 30.7%), followed by quionolones (239 million kg-days, 20.0%) and cephalosporines (200 million kg-days, 16.7%).

### Antimicrobial sales amount for oral use in pigs in 2018

In terms of the weight of active ingredient, tetracyclines represented the largest proportion (197,996kg, 45.3%) of the total usage, followed by sulfonamides (50,301kg, 13.6%) and penicillins (40,812kg, 9.3%). In terms of the number of DDDjps, tetracyclines represented the largest proportion (21,850 million kg-days, 28.8%) of the total usage, followed by sulfonamides (10,830 million kg-days, 14.3%) and amphenicols (8,867 million kg-days, 11.7%). In terms of the number of DDDvets, tetracyclines represented the largest proportion (12,112 million kg-days, 35.9%), followed by pleuromutillins (3,833 million kg-days, 11.4%) and macrolides (2,282 million kg-days, 8.5%).

### Comparison between the number of Japanese and European defined daily doses

**Fig 2:**
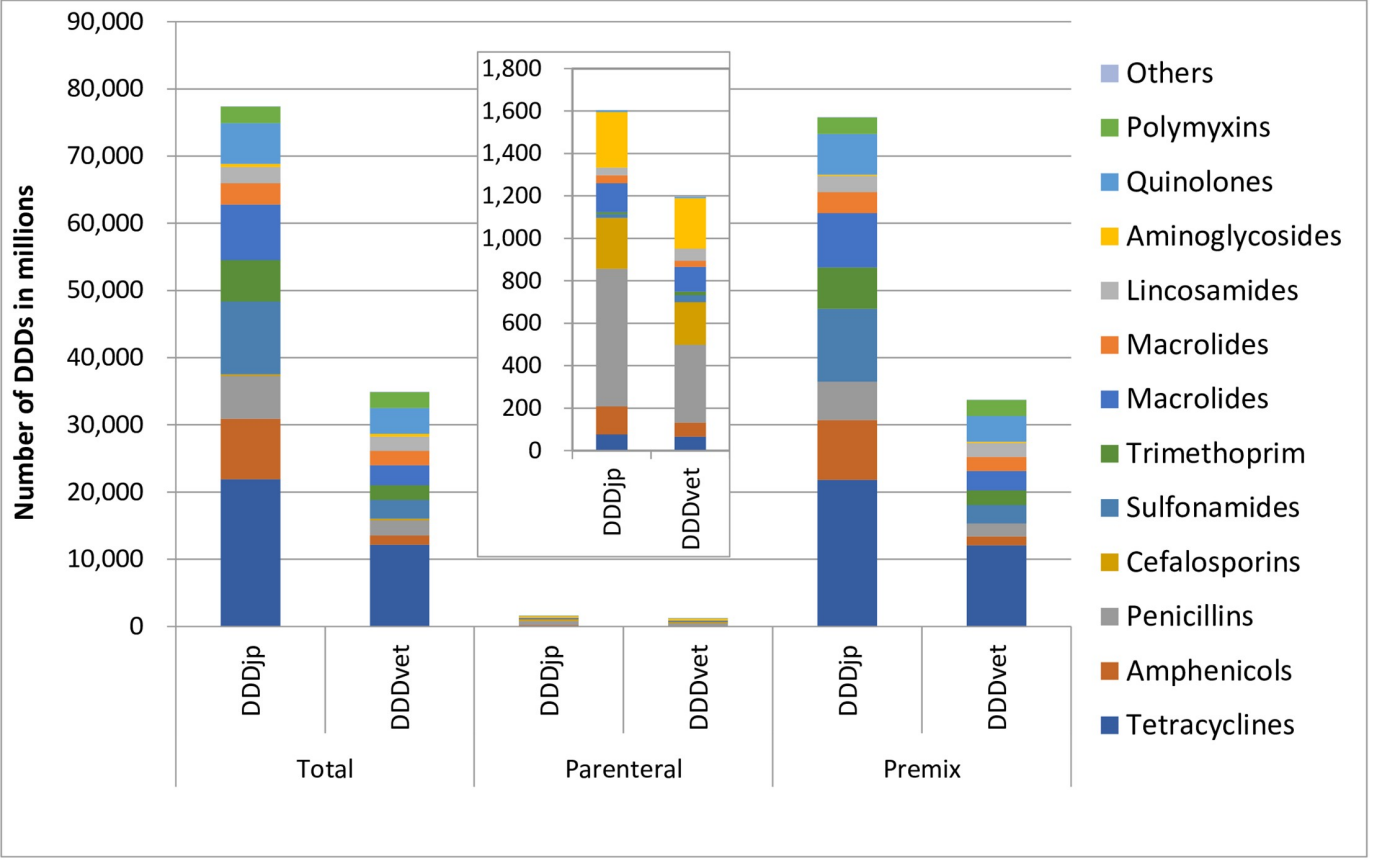
Comparison of number of Defined Daily Doses (DDD) of antimicrobial sales in the pig sector in Japan in 2018 calculated using DDDjp and DDDvet values. Inset shows the details of parenteral antimicrobial sales.

**Fig 3:**
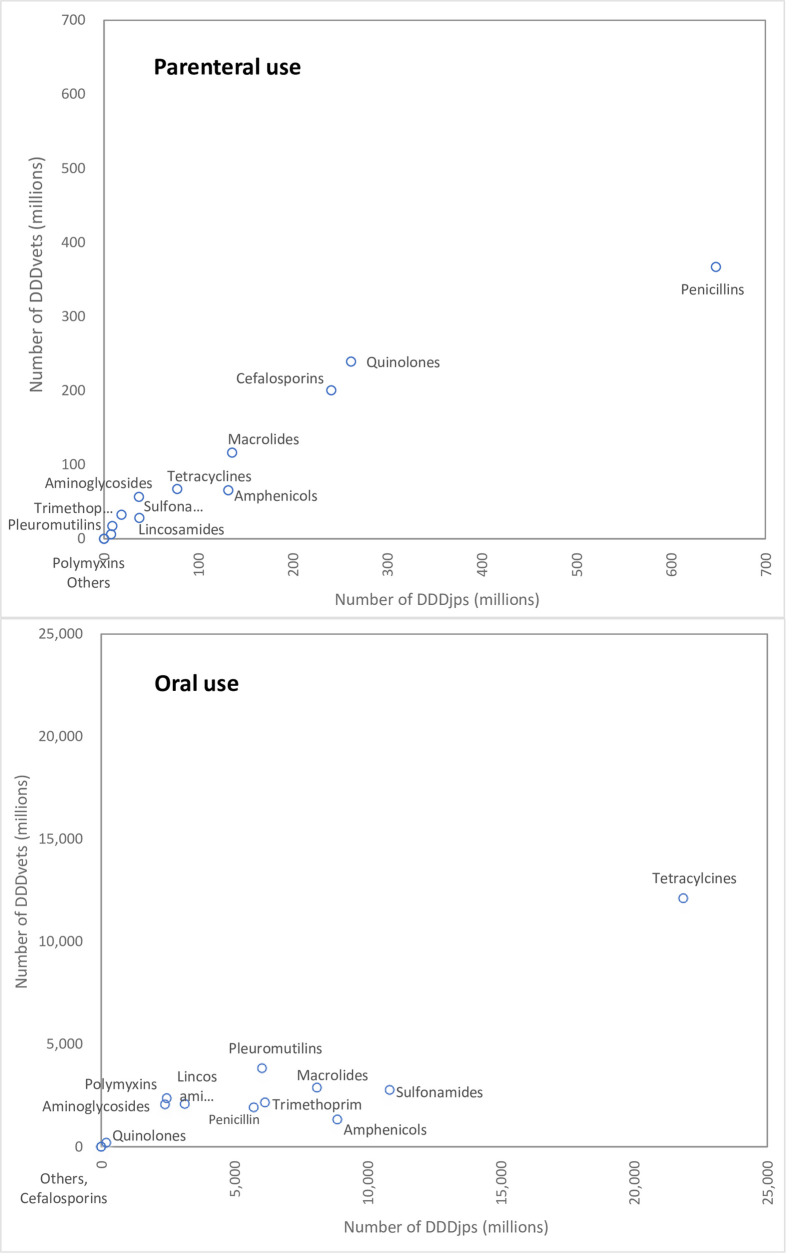
Scatterplots of the number of Defined Daily Doses (DDDs) for different antimicrobial classes calculated by Japanese values (DDDjp) and European values (DDDvet). Each open circle represents an antimicrobial class.

The number of DDDjps of antimicrobials sold for parenteral use was 1.34 times greater than that of DDDvets. The number of DDDjps of antimicrobials sold for oral use was 2.25 times greater than that of DDDvets (Table 2 and Fig 2). With regard to the number of DDDs of antimicrobial agents sold for parenteral use, the number of DDDjps was larger than DDDvets for most of the antimicrobial classes except for sulfonamides, trimethoprim and amynoglicodes (Fig 3: top). Spearman’s Rho test revealed that these two variables were significantly correlated (r = 0.978, *p*<0.001). The number of DDDjps sold for oral use was larger than DDDvets for all antimicrobial classes (Fig 3: bottom). Again, Spearman’s Rho test showed these two variables to be significantly correlated (r = 0.736, *p*<0.041).

### Temporal change of antimicrobial sales amount using different metrics

**Fig 4:**
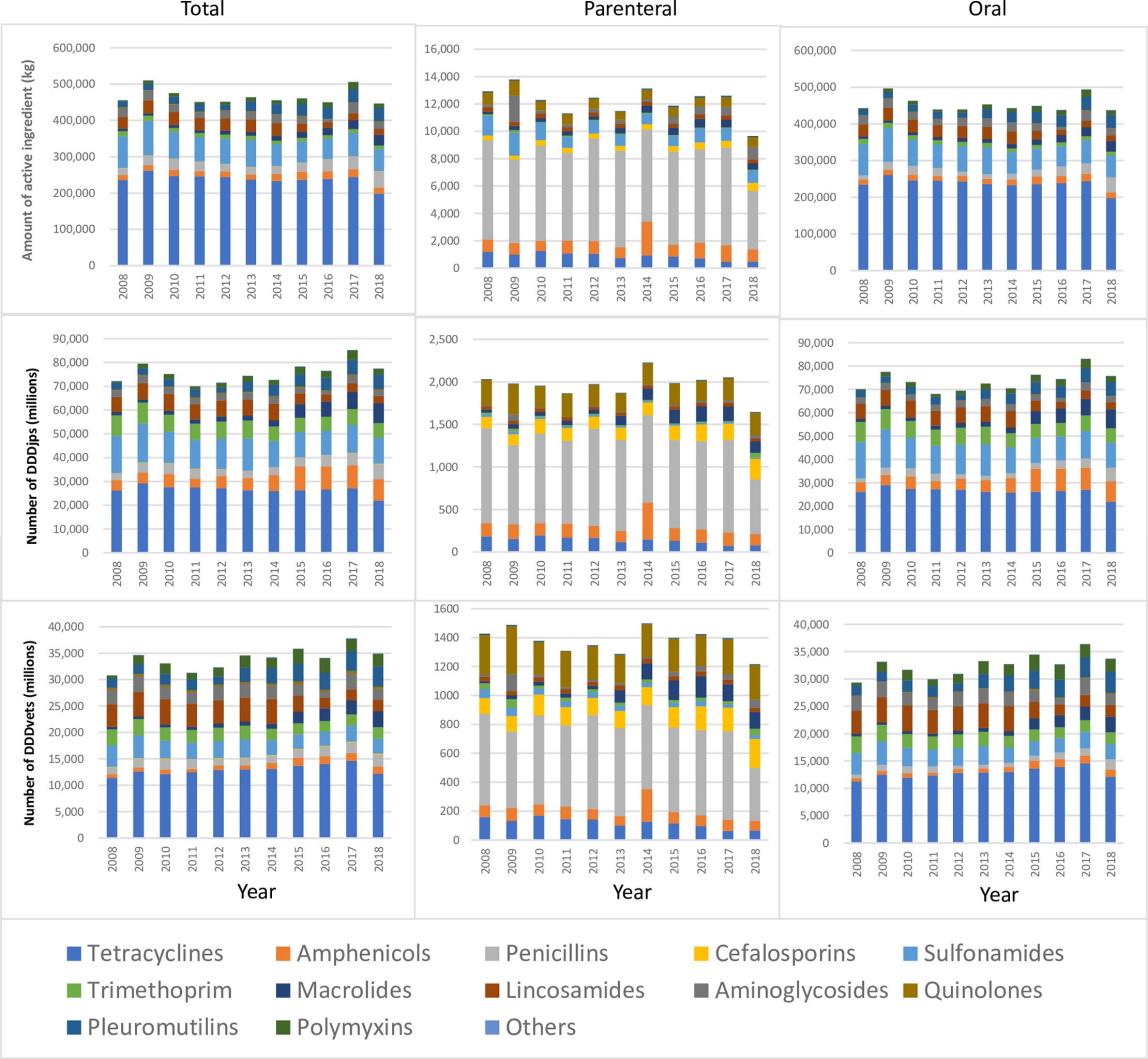
Evolution of antimicrobial sales for use in pigs in Japan from 2008 to 2018 in terms of weight of active ingredient (top row), number of Japanese Defined Daily Doses (DDDjps) (middle row) and number of European Defined Daily Doses (DDDvets) (bottom row).

The evolution of antimicrobial sales from 2008 to 2018 in terms of the weight of active ingredient, the number of DDDjps and the number of DDDvets are presented in Fig 4. The temporal changes between years saw the same trend regardless of the metrics used, except for between 2008 and 2009 when the weight of active ingredient sold for parenteral use increased and the corresponding number of DDDjps decreased. Between 2011 and 2012, the weight of active ingredient sold for oral use decreased while the numbers of DDDjps and DDDvets increased.

## Discussion

This study is the first attempt to evaluate the national antimicrobial sales amount in Japan using the number of DDDs. Dosage-based indicators have been used mainly to measure the antimicrobial use at farm level [13, 14, 21–23], except in France where a dosage-based indicator, ALEA (animal level of exposure for antimicrobials) was developed to monitor the antimicrobial use using national sales data [7].

### Effect of using a dosage-based indicator

The relative distribution by antimicrobial class differed depending on the metrics used. As a result, the temporal change pattern was reversed for certain years depending on the metrics used (between 2008 and 2009, the weight of active ingredient sold for parenteral use increased while the corresponding number of DDDjps decreased; between 2011 and 2012, the weight of active ingredient sold for oral use decreased while the numbers of DDDjps and DDDvets increased). In particular, most tetracyclines and sulfonamides have a dosage larger than other antimicrobial agents, resulting in that the relative distribution of these classes was large when measured by the weight of active ingredient but is lower when a dosage-based metric was used. On the contrary, high potency antimicrobials such as cephalosporins, macrolides and quinolons (for parenteral use) and amphenicols, macrolides, lincosamides and trimethoprims (for oral use) presented a larger relative distribution of these classes when a dosage-based metric was used. This change caused by the use of dosage-based indicator instead of using an indicator based on the weight of active ingredient has been highlighted in other studies [24–26]. This illustrates that the weight of active ingredient does not reflect treatment intensity and risk of development of antimicrobial resistance. Thus, the use of dosage-based metric is recommended for monitoring of antimicrobial use in pigs in Japan.

### Comparison between the number of DDDjps and the number DDDvets

This study shows that evaluating antimicrobial use at national level leads to a significant difference in the number of DDDs depending on whether the DDDjp or DDDvet values are used. The number of DDDjps was greater than the number of DDDvets for most antimicrobial classes. This was attributed to the fact that DDDjp values are lower than DDDvet values for most antimicrobial agents. Despite the fact that the number of DDDjps and the number of DDDvets calculated for different antimicrobial classes resulted in a positive correlated association, there are still deviations in the assessment of the various active ingredient classes and different administration routes.

Canada also found that in developing their country-specific DDD values, the majority of their DDD values were lower than their corresponding DDDvet values [27]. There are many reasons for the difference observed between DDDvet and DDDjp or DDD values in other non-European countries. One reason is that the EMA might have had a wider range of antimicrobial doses to work with due to the collection of antimicrobial agent doses from nine European countries [28, 29]. The different labelling regulations, different treatment indications and different husbandry practices might all contribute to the variations in DDDvet and DDDjp values. However, fully elucidating the reasons for these differences is beyond the scope of this study.

### Need for the use of Japanese Defined Daily Doses (DDDjp)

This study revealed that despite the large difference in the number of DDDjps and the number of DDDvets, a possible national level antimicrobial use monitoring system will provide similar conclusions regardless of whether the Japanese or European DDD value is used. Furthermore, this study showed that DDDvets did not cover all the antimicrobial agents used in veterinary medicine in Japan. Although drawing conclusions from differences between assigned DDDjp and DDDvet values is difficult as discussed previously, the differences in the number of DDDjps and DDDvets appear to confirm the need for Japanese DDDs, which are based on national approvals in comparison to the average EMA definitions collected from nine EU members and they better reflect antimicrobial selection pressure in the Japanese context.

### Limitations

Given the fact that the present study used calculations from the national sales data and DDD values based on national approvals, one should keep in mind that the exact amount of biomass subjected to antimicrobial treatment in terms of kg-days cannot be assessed because both over-dosing and under-dosing could alter the results. The calculation presented in this study only allows a statistical estimation of probable antimicrobial use but provides a consistent and transparent technical method for adjusting weight-based measures by dose. To avoid over- or under-estimation of antimicrobial use, the use of used daily doses (UDDs) might be a solution, but to assign the UDDs, additional information (such as the number of animals treated and the treatment duration) are required [30, 31].
